# Biological activity of tumor-treating fields in preclinical glioma models

**DOI:** 10.1038/cddis.2017.171

**Published:** 2017-04-20

**Authors:** Manuela Silginer, Michael Weller, Roger Stupp, Patrick Roth

**Affiliations:** 1 Laboratory of Molecular Neuro-Oncology, Department of Neurology, University Hospital Zurich and University of Zurich, Zurich, Switzerland; 2 Department of Oncology, University Hospital Zurich and University of Zurich, Zurich, Switzerland

## Abstract

Glioblastoma is the most common and aggressive form of intrinsic brain tumor with a very poor prognosis. Thus, novel therapeutic approaches are urgently needed. Tumor-treating fields (TTFields) may represent such a novel treatment option. The aim of this study was to investigate the effects of TTFields on glioma cells, as well as the functional characterization of the underlying mechanisms. Here, we assessed the anti-glioma activity of TTFields in several preclinical models. Applying TTFields resulted in the induction of cell death in a frequency- and intensity-dependent manner in long-term glioma cell lines, as well as glioma-initiating cells. Cell death occurred in the absence of caspase activation, but involved autophagy and necroptosis. Severe alterations in cell cycle progression and aberrant mitotic features, such as poly- and micronucleation, preceded the induction of cell death. Furthermore, exposure to TTFields led to reduced migration and invasion, which are both biological hallmarks of glioma cells. The combination of TTFields with irradiation or the alkylating agent, temozolomide (TMZ), resulted in additive or synergistic effects, and the O^6^-methyl-guanine DNA methyltransferase status did not influence the efficacy of TTFields. Importantly, TMZ-resistant glioma cells were responsive to TTFields application, highlighting the clinical potential of this therapeutic approach. In summary, our results indicate that TTFields induce autophagy, as well as necroptosis and hamper the migration and invasiveness of glioma cells. These findings may allow for a more detailed clinical evaluation of TTFields beyond the clinical data available so far.

Glioblastomas are among the most lethal neoplasms. Despite multimodal therapy, including maximal safe surgical resection followed by radio- and chemotherapy, the median survival is limited to approximately 16 months in selected patient populations.^1^ The highly invasive phenotype of these tumors precludes complete surgical resection and limits the efficacy of other local therapies. Moreover, defects in the apoptotic machinery of glioma cells account for their resistance to irradiation and chemotherapy. Therefore, novel therapeutic approaches are urgently needed.

Tumor-treating fields (TTFields) represent a novel treatment option for glioblastoma by creating alternating electric fields delivered through transducer arrays directly applied onto the scalp of patients. TTFields at intermediate frequencies between 10 kHz and 1 MHz circumvent the stimulation of excitable tissues such as nerves or muscles and do not induce tissue heating.^2^ TTFields are supposed to exert antitumor effects by targeting dividing tumor cells while sparing other cells in the brain that are not undergoing division.^3, 4^ At the beginning of mitosis, the electric field in a cell is mostly uniform, thus oscillating electric forces yield minimal movement on charged molecules and dipoles. Thereby TTFields may prevent tubulin subunits to attain correct orientation to build the mitotic spindle apparatus, so that mitosis becomes arrested. In contrast, during cytokinesis the electric field is non-uniform with the highest field intensity at the furrow that is connecting the two forming daughter cells. TTFields may therefore disturb the internal cell structure by causing polarized molecules and organelles to move toward the furrow, finally resulting in cell death.^2, 5^ Moreover, preliminary data suggest that TTFields may also exert immune-modulating effects.^6^ Thus, there are various hypotheses regarding the mechanisms that may contribute to the effectiveness of TTFields.

TTFields have been assessed in two phase III trials in patients with newly diagnosed, as well as recurrent glioblastoma. In patients with recurrent glioblastoma, TTFields had a comparable efficacy as that seen with a 'physician's best choice' chemotherapy regimen while having less toxicity.^7^ The results of a randomized phase III trial in patients with newly diagnosed glioblastoma suggest that the addition of TTFields to maintenance temozolomide (TMZ) chemotherapy prolongs progression-free and overall survival.^8^ Despite these encouraging data, TTFields are facing a lot of skepticism from patients and physicians, which, together with high treatment cost, has resulted in only limited use so far.^9, 10^ One of the major reasons causing low acceptance, has been the poor understanding of the underlying biology, which may explain the exact mechanism of action of alternating electric fields against tumors cells. The aim of the current project was to investigate the effects of TTFields on glioma cells including glioma-initiating cells (GICs), as well as the functional characterization of the underlying mechanisms.

## Results

### TTFields induce cell death in an intensity- and frequency-dependent manner in human glioma cells

**Figure 1:**
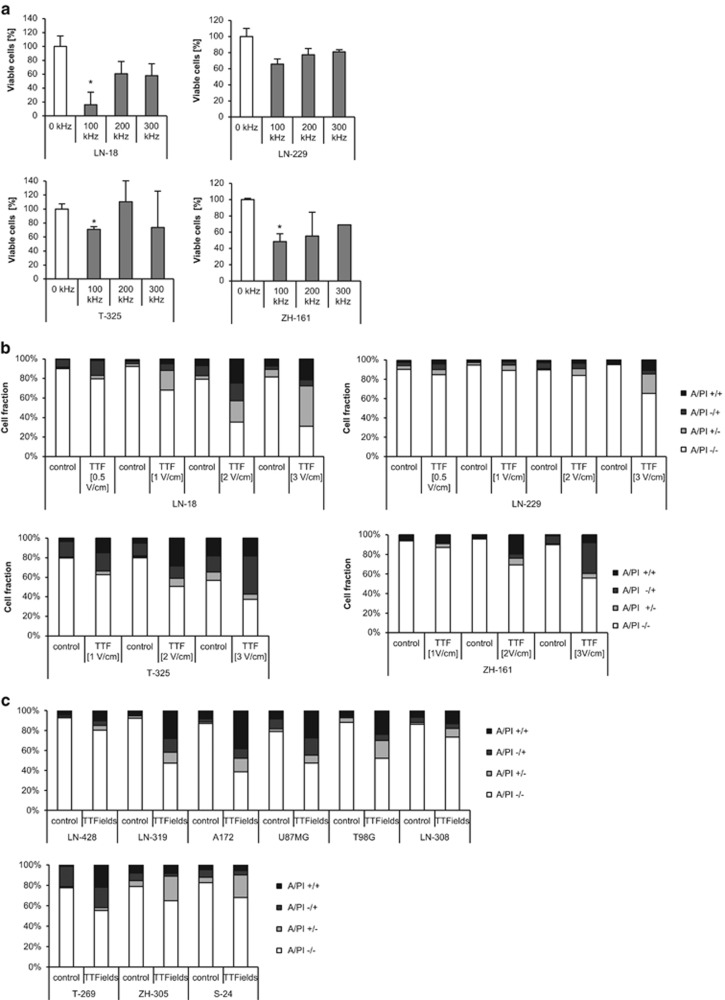
TTFields induce cell death in an intensity- and frequency-dependent manner in human glioma cells. (**a**) LN-18, LN-229, T-325 or ZH-161 cells were left untreated or exposed to TTFields (TTF; 2 V/cm) at the indicated frequencies for 48 h. Viable cell counts were obtained using trypan blue exclusion (*P<0.05). (**b**) The human LTC LN-18 or LN-229 (top) or T-325 or ZH-161 GIC (bottom) were exposed to increasing intensities of TTFields as indicated for 48 h (LTC) or 72 h (GIC). Cell death was assessed by annexin V/PI staining. (**c**) The LTC LN-428, LN-319, A172, U87MG, T98G or LN-308 (top) and the GIC T-269, S-24 or ZH-305 (bottom) were exposed to TTFields (2 V/cm) or not for 48 or 72 h, respectively, and subsequently analyzed for cell death by annexin V/PI staining

The human long-term glioma cell (LTC) LN-18 or LN-229 or the human GIC ZH-161 or T-325 were exposed to increasing frequencies of TTFields to define the optimal parameters for penetrating the cell membrane. Most prominent effects on cell viability were observed at 100 kHz (Figure 1a). This frequency was chosen for all further experiments. Next, LN-18, LN-229, T-325 or ZH-161 cells were exposed to increasing intensities of TTFields. Expectedly, TTFields induced cell death in an intensity-dependent manner, however, more extensively in LN-18 than in LN-229 cells, with a substantial proportion of annexin V-positive cells (Figure 1b, top, Supplementary Figure 1a). Similarly, TTFields exposure resulted in cell death also in the GIC lines ZH-161 and T-325 in an intensity-dependent manner (Figure 1b, bottom, Supplementary Figure 1b). Importantly, the whole panel of glioma cell lines, composed of LTC and GIC, was sensitive to TTFields treatment to a variable degree in terms of cell death induction (Figure 1c).

### TTField-induced cell death occurs in a caspase-independent manner

**Figure 2:**
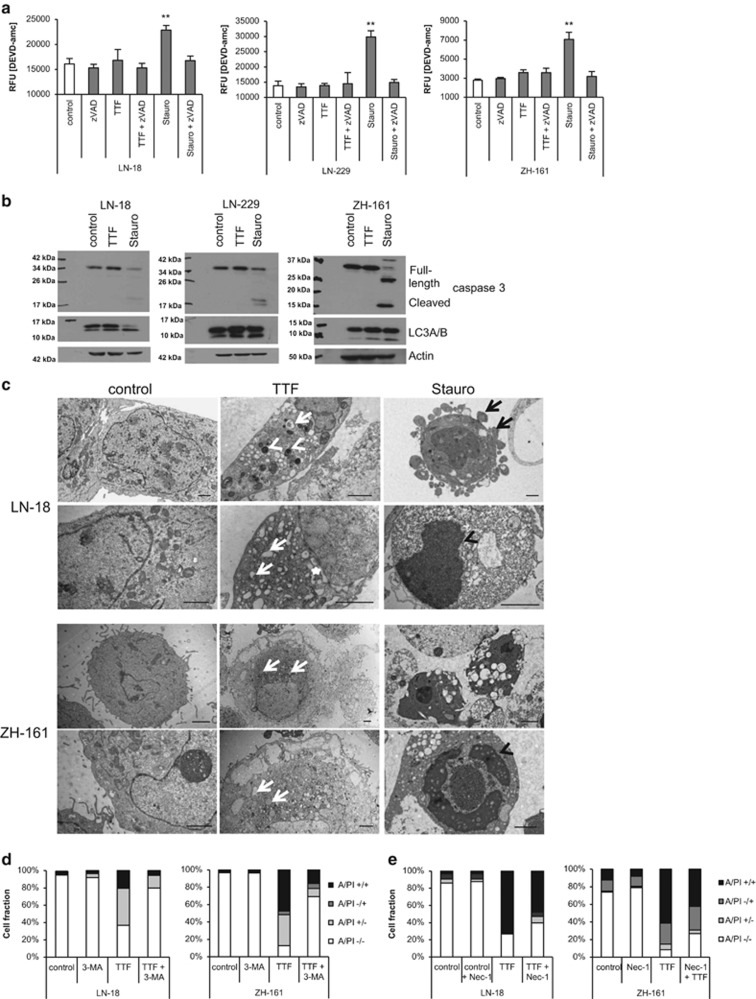
TTFields induce cell death via autophagy. (**a**) LN-18, LN-229 or ZH-161 cells were left untreated or exposed to TTFields (TTF, 3 V/cm), staurosporine (Stauro, 1 *μ*M), zVAD-fmk (10 *μ*M) or combinations thereof for 6 h. DEVD-amc cleaving activity was determined fluorometrically (**P<0.01). (**b**) Whole-cell lysates of LN-18, LN-229 or ZH-161 cells, untreated or exposed to TTF (3 V/cm) or staurosporine (1 *μ*M) for 48 h, were analyzed for full-length and cleaved caspase-3 and LC3A/B protein levels. Actin was used as a loading control. (**c**) LN-18 or ZH-161 cells were left untreated or exposed to TTFields (TTF, 3 V/cm) or Stauro (1 *μ*M) for 48 h and then monitored by electron microscopy. Autophagosomes are indicated with white arrows, white arrowheads point to mitochondria with swollen matrices, the white star to the dilated endoplasmatic reticulum, black arrows to membrane blebs and black arrowheads to condensed DNA along the nucleus (scale bar, 2 *μ*m). (**d**) LN-18 or ZH-161 cells were treated with 3-methyladenine (3-MA, 1 mM) for 60 min followed by TTFields (2 V/cm) or not for 72 h. Cell death was assessed by annexin V/PI staining. (**e**) LN-18 or ZH-161 cells were treated with necrostatin-1 (Nec-1; 100 *μ*M) for 60 min followed by TTFields (3 V/cm) or not for 72 h. Cell death was assessed by annexin V/PI staining

Next, we aimed at assessing the mode of cell death upon TTFields treatment in more detail. Increased caspase activity, as determined by DEVD-amc cleavage, was detected in cells treated with staurosporine, a prototypic inducer of canonical, caspase-dependent apoptosis, but not upon exposure to TTFields. Accordingly, the broad spectrum caspase inhibitor, zVAD-fmk, inhibited caspase activity induced by staurosporine, but had no effect in TTField-treated cells (Figure 2a). Furthermore, caspase-3 processing was not detected upon exposure to TTFields, indicating that cell death induced by TTField occurs in a caspase-independent manner. Interestingly, when LN-18, LN-229 or ZH-161 cells were exposed to TTFields or staurosporine, increased LC3A/B-II levels, an indicator for the induction of autophagy, were observed (Figure 2b,Supplementary Figure 2c). We therefore went on to examine the morphology of TTField-induced cell death using transmission electron microscopy. TTField-exposed LN-18 or ZH-161 cells showed typical signs of autophagy such as a markedly increased frequency of autophagosomes, mitochondria with swollen matrices or a dilated endoplasmatic reticulum. In contrast, staurosporine, as a classical inducer of apoptosis, led to membrane blebbing or DNA condensation along the nucleus and dark cytoplasm (Figure 2c). To clarify whether autophagy in the context of TTFields acts as a survival or cell death signal, glioma cells were cultured in the absence or presence of the autophagy inhibitor 3-methyladenine. Blocking autophagy reduced the amount of dead cells upon TTFields exposure, suggesting that autophagy mediates TTField-induced cell death (Figure 2d, Supplementary Figure 2a). In addition, when cells were co-treated with the receptor-interacting protein-1 inhibitor necrostatin-1, which blocks necroptosis, cell death induced by TTFields was partly attenuated, suggesting that necroptosis also contributes to cell death upon TTFields administration (Figure 2e, Supplementary Figure 2B).

### TTFields interfere with cell cycle progression

**Figure 3:**
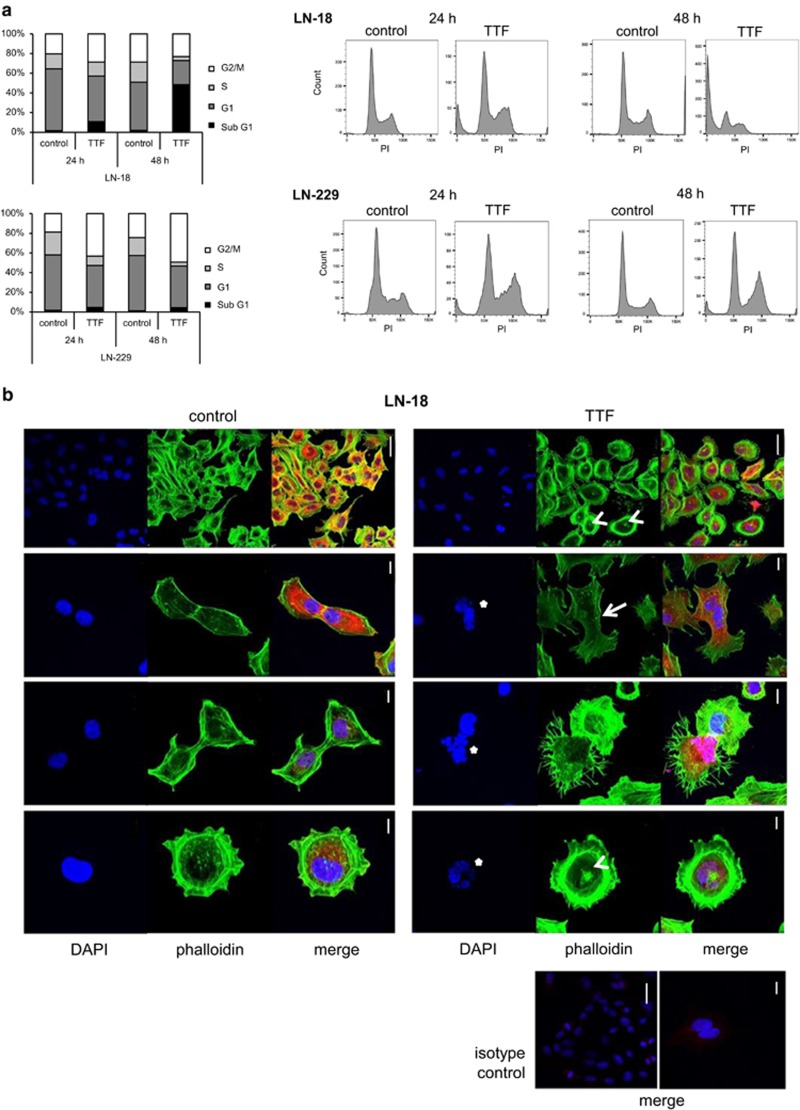
TTFields interfere with cell cycle progression. (**a**) LN-18 or LN-229 cells were exposed to TTFields (2 V/cm) for 24 or 48 h as indicated. Cell cycle analysis was performed by flow cytometry and cell cycle distribution is shown in bar graphs, as well as flow cytometry profiles. (**b**) LN-18 cells were left untreated or exposed to TTFields (2 V/cm) for 24 h. Actin (phalloidin staining, green), beta III tubulin (red) and nuclei (DAPI, blue) were analyzed by immunofluorescence. Different magnifications are shown. An isotype control for anti-beta III tubulin is included at the bottom. Accumulations of nuclear actin are indicated with arrows, arrowheads point to altered cell shape and stars to nuclear aberrations (scale bar, 10 *μ*m)

Next, we performed cell cycle analyses, which demonstrated severe alterations that may ultimately result in cell death. In fact, we observed an increased number of cells in the G2/M phase after 24 h, followed by an accumulation of cells in the sub-G1 phase after 48 h of treatment with TTFields (Figure 3a). In line with these findings, confocal microscopy revealed an increased number of abnormal mitotic events in TTField-treated cells such as poly- or micronucleation. In addition, many cells exhibited aberrant changes in cellular shape and accumulations of actin in the nucleus, which are further indicators of cellular stress (Figure 3b).

### TTFields reduce migration and invasion of glioma cells

**Figure 4:**
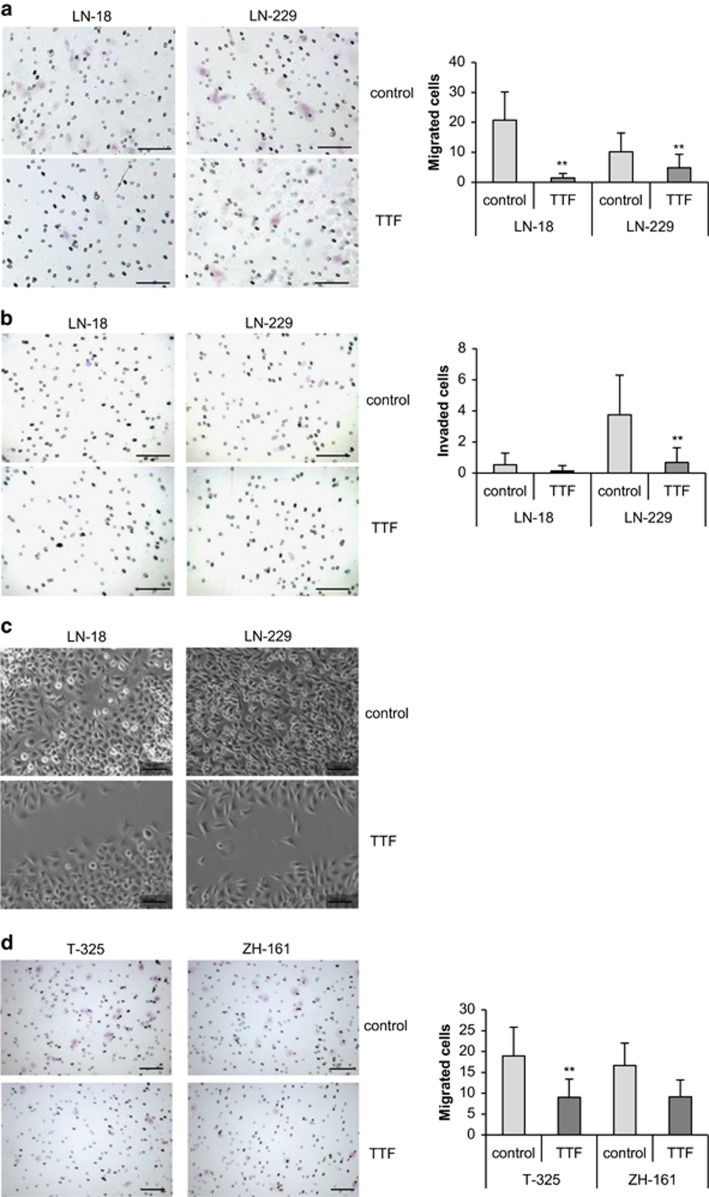
TTFields reduce migration and invasion of glioma cells. (**a** and **b**) LN-18 or LN-229 cells were left untreated or exposed to TTFields (2 V/cm) for 24 h. Subsequently, modified migration/matrigel invasion Boyden chamber assays were performed to analyze migration (**a**) or invasion (**b**). Data are expressed as mean cells per field of vision (FoV) and exemplary photographs of migrated or invaded LN-18 or LN-229 cells are shown (scale bar, 100 *μ*m). (**c**) A scratch wound-healing assay was performed during which LN-18 or LN-229 cells were left untreated or exposed to TTFields (2 V/cm). After 24 h, the gap distance was imaged on a microscope and representative images are shown (scale bar, 100 *μ*m). (**d**) T-325 or ZH-161 cells were left untreated or exposed to TTFields (2 V/cm) for 24 h. Subsequently, a modified migration Boyden chamber assay was performed. Data are expressed as mean cells per FoV and exemplary photographs of migrated cells preexposed to TTFields or not are shown (scale bar, 100 *μ*m). (**P<0.01)

As glioma cells are characterized by their ability to migrate and invade into the surrounding healthy tissue, we asked whether TTFields modulate their migration and invasiveness. We observed impaired migration and invasiveness of LN-18 and LN-229 cells upon exposure to TTFields using transwell migration and Matrigel invasion assays (Figures 4a and b). Importantly, the effects on migration and invasion exceeded those predicted from the loss of viable cells at the same time (Supplementary Figure 3a). These data were further corroborated using a scratch wound-healing assay, which showed reduced migration and growth toward the center of the gap of TTField-exposed LN-18 or LN-229 cells (Figure 4c). Importantly, TTFields also interfered with the migration of the GIC lines T-325 and ZH-161, which are thought to be of particular importance for tumorigenesis and tumor recurrence (Figure 4d). Again, the effects on migration of GIC were more prominent than those expected from loss of viability (Supplementary Figure 3b).

### TTFields act in an additive or synergistic manner with irradiation or TMZ

**Figure 5:**
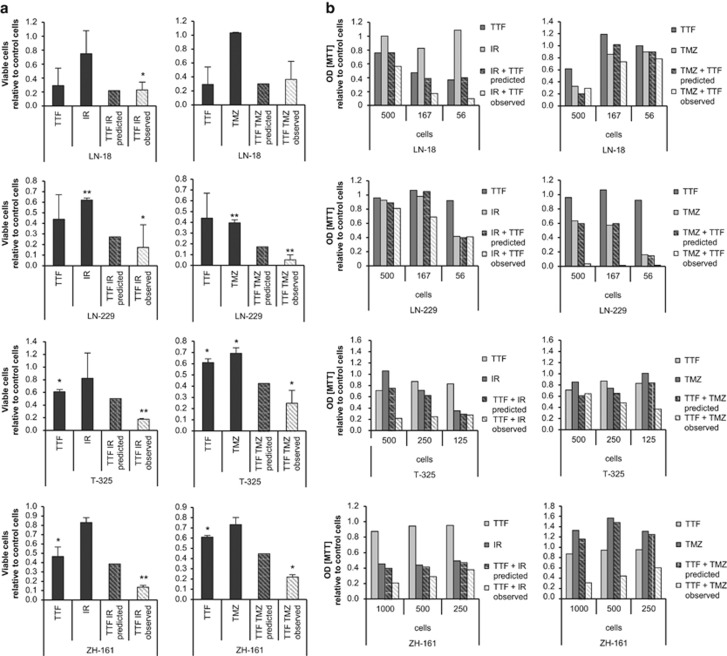
TTFields act synergistically with irradiation or TMZ to reduce acute and clonogenic survival. (**a**) LN-18, LN-229, T-325 or ZH-161 cells were left untreated or irradiated (LN-18, LN-229, 3 Gy; T-325 and ZH-161, 5 Gy) followed or not by TTFields (2 V/cm) for 24 h (left panel). Alternatively, the cells were exposed to DMSO control or TMZ (LN-18, 100 *μ*M; LN-229, 5 *μ*M; T-325, 200 *μ*M; ZH-161, 25 *μ*M) paralleled by TTFields exposure (2 V/cm) for 24 h or not (right panel). After another 48 h, viable cells were counted by Trypan blue exclusion test (*P<0.05; **P<0.01, relative to control cells). (**b**) Cells, treated as in **a**, were seeded at the indicated densities and clonogenic survival was assessed by 3-(4,5-dimethylthiazol-2-yl)-2,5-diphenyltetrazolium bromide (MTT) assay after 14 days

As radiation therapy and alkylating chemotherapy with TMZ are part of the standard treatment for glioblastoma patients, we assessed the activity of TTFields in combination with these treatment modalities. In acute cytotoxicity assays, mostly additive effects were observed in LN-18 cells when TTFields were combined with TMZ or irradiation, whereas synergistic effects were seen in LN-229, T-325 or ZH-161 cells by either combination (Figure 5a). Analyses of clonogenic survival in limiting dilution assays revealed synergistic effects of TTFields in combination with irradiation in LN-18, as well as T-325 cells, and with TMZ in LN-229 and ZH-161 cells (Figure 5b). Hence, depending on the cell line, the combination approach and the read-out, the effects may be additive or synergistic, however, never antagonistic in the models studied here.

**Figure 6:**
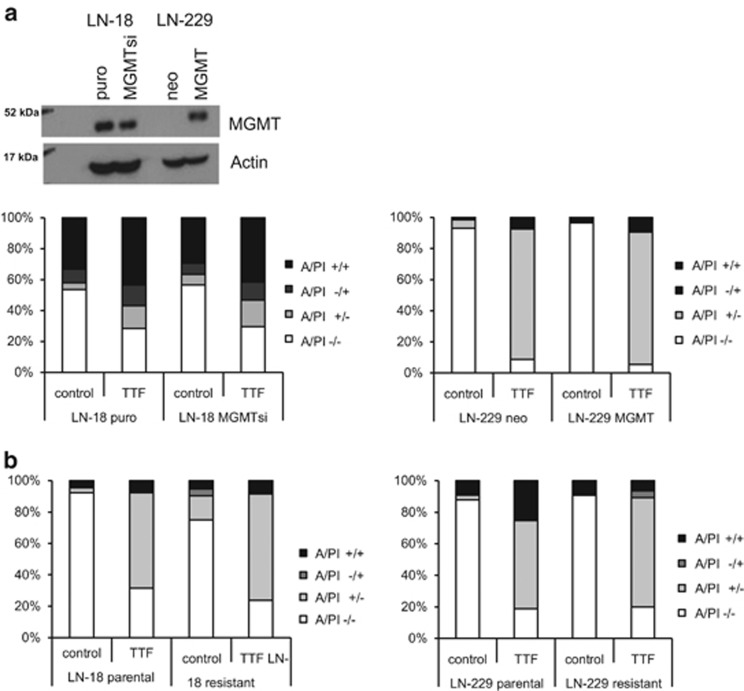
TTFields induce cell death in a MGMT-independent manner. (**a**) Whole-cell lysates of LN-18 puro or MGMTsi, or LN-229 neo or MGMT cells were analyzed for MGMT expression by immunoblot. Actin was used as a control (top). The cells were exposed to TTFields (2 V/cm) or not for 48 h and analyzed for cell death by annexin V/PI staining. (**b**) LN-18 parental or TMZ-resistant, or LN-229 parental or TMZ-resistant cells were exposed to TTFields (2 V/cm) or not. After 48 h cell death was analyzed by annexin V/PI staining

It is well known that the DNA repair protein O^6^-methyl-guanine DNA methyltransferase (MGMT) influences the antitumor activity of the alkylating agent, TMZ, *in vivo* and *in vitro*.^11, 12^ Thus, we determined a possible impact of the cells' MGMT status on their sensitivity to TTFields. To this end, we used genetically engineered cells with modulated MGMT expression. LN-18 cells with a silenced *MGMT* gene or LN-229 cells overexpressing *MGMT* displayed similar sensitivity to TTFields as their wild-type counterparts. Thus, TTField-induced cell death seems to be independent from the MGMT status (Figure 6a, Supplementary Figure 3c). Virtually all patients relapse or progress during or after TMZ therapy, which encouraged us to analyze whether TTFields exert antitumor activity against TMZ-resistant glioma cells, which had been generated by repeated exposure to TMZ.^13^ TMZ-resistant LN-18 or LN-229 cells were similarly sensitive to TTField-induced cell death as their parental counterparts (Figure 6b), which does not point to mechanisms of cross-resistance.

## Discussion

Glioblastoma is a highly lethal brain tumor with a median overall survival of approximately 16 months within clinical trials. Since 2005, the standard of care for newly diagnosed glioblastoma has been maximal safe tumor resection, followed by irradiation with concomitant and maintenance chemotherapy with TMZ. However, the survival benefit conferred by TMZ is largely restricted to patients with tumors that harbor a methylation of the *MGMT* gene promoter.^12, 14^ TTFields represent a novel treatment approach that has already been approved by FDA for newly diagnosed, as well as recurrent glioblastoma based on the results of two phase III trials.^7, 8^ However, there is a mismatch between the encouraging clinical data and the biological effects of TTFields on tumor cells, which have only been poorly understood.

Here we demonstrate that TTFields potently induce cell death in all glioma cell lines. Importantly, TTFields treatment displayed strong activity against glioma cells with stem-like properties, too, which is of particular interest because of the potential contribution of this cell population to tumor recurrence and therapy resistance (Figure 1).^15, 16^ Glioblastomas are characterized by extensive heterogeneity at a cellular and molecular level.^17, 18^ Owing to advances in single-cell technology it became clear that each tumor contains multiple distinct populations of tumor cells with variation in the expression of a range of transcriptional programs, including oncogenic signaling, proliferation, immune response and hypoxia.^19^ Hence, inter- and intra-tumoral heterogeneity may contribute to the failure of targeted therapeutic treatment strategies, which greatly depend on the activity of certain molecular pathways.^20^ As TTFields are a physical rather than chemical treatment modality, they may be effective over a wider range of tumors with heterogeneous characteristics.^5^ The antitumor activity of TTFields may depend on parameters like the cell size that may determine the optimal frequency to induce cell death or the cell's doubling time as TTFields is believed to act mainly during cellular division.^5, 21^ Accordingly, GIC may be less responsive to TTFields than other tumor cells because of slower proliferation and higher heterogeneity in cell size.

In contrast to a report suggesting that HCT-116 colon cancer cells undergo apoptosis upon TTFields treatment,^22^ we did not observe caspase activation, a hallmark of apoptosis, in the cell lines tested in this study. However, our experiments revealed an important role for autophagy and necroptosis in TTField-induced cell death (Figure 2). Electron microscopy further supported the induction of autophagy, whereas no cellular changes typically associated with apoptosis were detected. Thus, the type of cell death induced upon TTFields treatment may differ between tumor entities and cell lines. In line with other studies, we observed an increased number of nuclear aberrations in TTField-treated cells that finally result in cell death (Figure 3).^22, 23^ Moreover, we noticed an accumulation of nuclear actin filaments, which has been attributed a consequence of cellular stress and may have a protective role.^24^

Glioblastoma is characterized by highly infiltrative growth, which precludes complete surgical resection and thus contributes to its aggressive phenotype.^25^ Upon exposure to TTFields, we found aberrant changes in the shape of glioma cells (Figure 3), which may indicate altered cell motility.^26^ Accordingly, we observed reduced migration and invasion of TTField-treated glioma cells (Figure 4). In line with these data, reduced metastatic spread of solid tumors to the lungs by TTFields treatment has been reported and may be a result of impaired formation of microtubule-based processes in migrating cells.^6, 26, 27^

The combination of TTFields with irradiation or TMZ showed additive or synergistic effects supporting the clinical combination of these treatment modalities as done for TMZ in one trial (Figure 5).^8^ Similarly, it has been reported that combining TTFields with drugs, such as paclitaxel or doxorubicin, may result in reduced tumor cell proliferation and viability in preclinical models.^28, 29^ In our experiments, altering MGMT expression did not influence the efficacy of TTFields, and TMZ-resistant glioma cells remained responsive to TTFields treatment suggesting that the mechanisms of resistance toward TMZ, which may involve MGMT but also other mediators^13^ do not overlap with a putative resistance to TTFields (Figure 6). Accordingly, TTFields may be particularly attractive for the majority of glioblastoma patients with tumors that are unlikely to benefit from TMZ treatment because of the unmethylated MGMT promoter. These findings are in line with data demonstrating that TTFields are also active against mitoxantrone- or doxorubicin-resistant breast cancer cells that overexpress ABC transporters. Moreover, it was shown that TTFields re-sensitizes drug-resistant tumor cells to chemotherapeutic agents, a finding that was interpreted as TTField-induced changes on the integrity of the cytoskeleton and microtubules, as well as mitochondria distribution.^29^

In summary, TTFields represent a novel therapeutic approach that has entered late-stage clinical development in glioblastoma patients. Our data set reveals that TTFields induce autophagy and necroptosis and interfere with the migration and invasion of glioma cells *in vitro*. The additive or synergistic effects of TTFields in combination with irradiation or TMZ, as well as their ability to induce cell death in TMZ-resistant glioma cells may represent a biological rationale for the further clinical evaluation of TTFields in glioma patients.

## Materials and Methods

### Cells and reagents

The human LTC lines LN-18, LN-229, LN-428, LN-319, A172 and LN-308 were kindly provided by Dr. N de Tribolet (Lausanne, Switzerland) and U87MG and T98G were obtained from the American Type Culture Collection (Manassas, VA, USA). The generation of LN-229 cells overexpressing MGMT and LN-18 cells with a silenced *MGMT* gene has been described.^11, 30^ Cells were cultured in Dulbecco's modified Eagle's medium (DMEM; Invitrogen, Basel, Switzerland), containing 2 mM l-glutamine (Gibco Life Technologies, Paisley, UK), penicillin (100 IU/ml)/streptomycin (100 *μ*g/ml) (Sigma-Aldrich, St. Louis, MO, USA) and 10% fetal calf serum (PAA, Vienna, Austria). The GIC lines T-269, T-325, S-24, ZH-161 and ZH-305 were established from freshly resected tumors and cultured in phenol red-free neurobasal medium with B-27 supplement (20 *μ*l/ml), glutamax (10 *μ*l/ml) (Invitrogen), fibroblast growth factor-2 and epidermal growth factor (20 ng/ml each; Peprotech, Rocky Hill, PA, USA).^31^ Cells were authenticated routinely at the Leibniz Institute DSMZ-German Collection of Microorganisms and Cell Cultures, Braunschweig, Germany by short tandem repeat analysis, LTC lastly in 2013 and GIC in 2016. Accutase was purchased from PAA. The following antibodies were used for immunoblot analysis: caspase-3 antibody and LC3A/B antibodies were obtained from Cell Signaling Technology (Boston, MA, USA), anti-MGMT antibody from Alpha Diagnostics (San Antonio, TX, USA) and goat polyclonal antibody to actin was obtained from Santa Cruz Biotechnology (Santa Cruz, CA, USA). zVAD-fmk was purchased from Tocris Bioscience (Bristol, UK), 3-methyladenine from Sigma-Aldrich, staurosporine from AppliChem (Darmstadt, Germany) and necrostatin-1 from Merck KGaA (Darmstadt, Germany). TMZ was provided by Merck & Co (Whitehouse Station, NJ, USA). Irradiation of cells was performed in a co-radiation source (Gebrüder Sulzer, Thermische Energiesysteme, 60-Co, Winterthur, Switzerland).

### TTFields treatment

To study the effects of TTFields on cell cultures *in vitro*, we used the inovitro system, a laboratory research device provided by Novocure (Haifa, Israel). A cell suspension containing 20 000 cells in 200 *μ*l medium was seeded as a drop in the center of a glass coverslip (20 mm diameter; Thermo Scientific, Waltham, MA, USA) within a ceramic dish. After attachment of the cells, 2 ml of medium were added and the ceramic dishes were placed onto a base plate connected to two pairs of electrodes perpendicular to each other and linked to a sinusoid function generator and an amplifier in order to generate alternating electric fields. The electric field intensities are expressed in V/cm root mean square.

### Migration and invasion

A cell suspension containing 40 000 or 80 000 viable cells pretreated with TTFields or not for 24 h before migration or invasion, respectively, was added to the upper well of transwell migration inserts or to BD BioCoatTM MatrigelTM invasion chambers (pore size: 8 *μ*m, BD Biosciences, Franklin Lakes, NJ, USA). In the lower well, 700 *μ*l of NIH-3T3 cell-derived conditioned medium were used as a chemoattractant. After 16 h at 37 °C and 5% CO_2_, the cells were fixed in ice-cold methanol for 10 min and stained with Mayer's alum hematoxylin for 60 min. Inserts were mounted in glass slides and nine fields per sample were counted on a microscope.

### Scratch wound-healing assay

Cells were seeded on Thermanox coverslips (Thermo Scientific). At confluency, two straight lines in perpendicular direction were scratched into the monolayer using a 1 ml pipette tip. Cells were washed twice to remove detached cells and then grown in serum-containing medium for additional 24 h in the absence or presence of TTFields. The gap distance was imaged on a microscope.

### Immunoblot analysis

Cells were lysed in radioimmunoprecipitation assay buffer (10 mM Tris pH 8.0, 150 mM NaCl, 1% NP-40, 0.5% deoxycholate, 0.1% sodium dodecyl sulfate) supplemented with 1x complete inhibitor mix (Roche Diagnostics, Grenzach-Wyhlen, Germany) and phosphatase inhibitor cocktails 1 and 2 (Sigma-Aldrich) and protein levels were analyzed by immunoblot using 30 *μ*g of protein per lane mixed with Laemmli buffer containing *β*-mercaptoethanol, using the respective antibodies. Protein bands were visualized using horseradish peroxidase-coupled secondary antibodies (Sigma-Aldrich) and enhanced chemiluminescence (Perbio, Bonn, Germany).^32^

### Viability assay

Cells, treated as indicated, were detached with accutase and resuspended in PBS. The number of viable cells was assessed by counting the cells with the Trypan blue dye exclusion method using a Vi-CELL Cell Viability Analyzer (Beckman Coulter, Nyon, Switzerland).

### Clonogenic survival assay

The cells were pretreated as indicated and then seeded at the indicated densities in 96-well plates, followed by observation for 7–14 days. As a surrogate marker of viability, metabolic activity was assessed using 3-(4,5-dimethylthiazol-2-yl)-2,5-diphenyltetrazolium bromide (Sigma-Aldrich).

### Annexin V/propidium iodide (PI) flow cytometry

For cell death analysis, cells were treated as indicated, followed by resuspension in annexin buffer (10 mM HEPES, 140 nM NaCl, 2.5 mM CaCl2, pH 7.4) and staining with Pacific blue-labeled annexin V from Biolegend (San Diego, CA, USA) and PI (Sigma-Aldrich) for 15 min at room temperature in the dark. Samples were analyzed by flow cytometry. Annexin V- or PI-positive cells were counted as dead cells (either apoptotic or necrotic), and the remaining cells were designated the surviving cell fraction.

### Cell cycle analysis

Cells were fixed in ice-cold 70% ethanol for 1 h on ice, subsequently washed, stained with a solution containing 0.5 mg/ml PI, 1 mg/ml RNase A (Sigma-Aldrich) and 0.1% Triton X-100 (Sigma-Aldrich) in PBS for 30 min at 4 °C, washed and then analyzed by flow cytometry.

### Caspase activity assay

Glioma cells, exposed to TTFields or not, were grown in phenol red-free medium. Subsequently, the cells were lysed and exposed to 12.5 *μ*M of the fluorescent substrate DEVD-amc (Bachem AG, Bubendorf, Switzerland) for 1 h. Caspase activity was assessed using a Mithras microplate reader (Berthold Technologies, Bad Wildbad, Germany).

### Immunofluorescence

Cells, grown on glass-coverslips, were exposed to TTFields or not for 24 h, and then fixed with 4% paraformaldehyde. Subsequently, the cells were washed with 0.1% Triton X-100 (Sigma-Aldrich), blocked in 5% BSA and incubated with anti-beta III tubulin antibody (Abcam, Cambridge, UK) (diluted 1:20) or isotype control overnight at 4 °C, followed by an incubation with a goat anti-rabbit IgG-Alexa Fluor 594-coupled secondary antibody (Invitrogen) (diluted 1:100) and nuclear DNA labeling with DAPI (Invitrogen). Finally, the cells were stained with Alexa Fluor 488 phalloidin (Thermo Scientific), diluted 1:20, at room temperature for 20 min. Images were acquired using a Leica SP5 confocal microscope (Wetzlar, Germany).

### Transmission electron microscopy

The cells including supernatant were fixed in a 2x fixation solution (2.5% glutaraldehyde and 1.6% formalaldehyde in a 100 mM sodium cacodylate buffer, pH 7.4, final concentration), dehydrated in a graded ethanol series and embedded into Epon. Sections of 60 nm were imaged with a Tecnai Spirit transmission electron microscope (FEI, Hillsborough, OR, USA).

### Statistical analysis

Data are presented as means and S.D. The experiments shown were commonly repeated three times. For some studies, representative experiments are shown. Analysis of significance was performed using a two-tailed Student's *t*-test (Excel, Microsoft, Redmond, WA, USA) (*P<0.05, **P<0.01).
