# Supplementary material for: Biological activity of tumor-treating fields in preclinical glioma models
Source: Cell Death Dis. 2017 Apr 20;8(4):e2753–. doi: 10.1038/cddis.2017.171 (PMC5477589; doi:10.1038/cddis.2017.171)
Supplement: Supplementary Figures [file cddis2017171x1.ppt]

## Slide 1
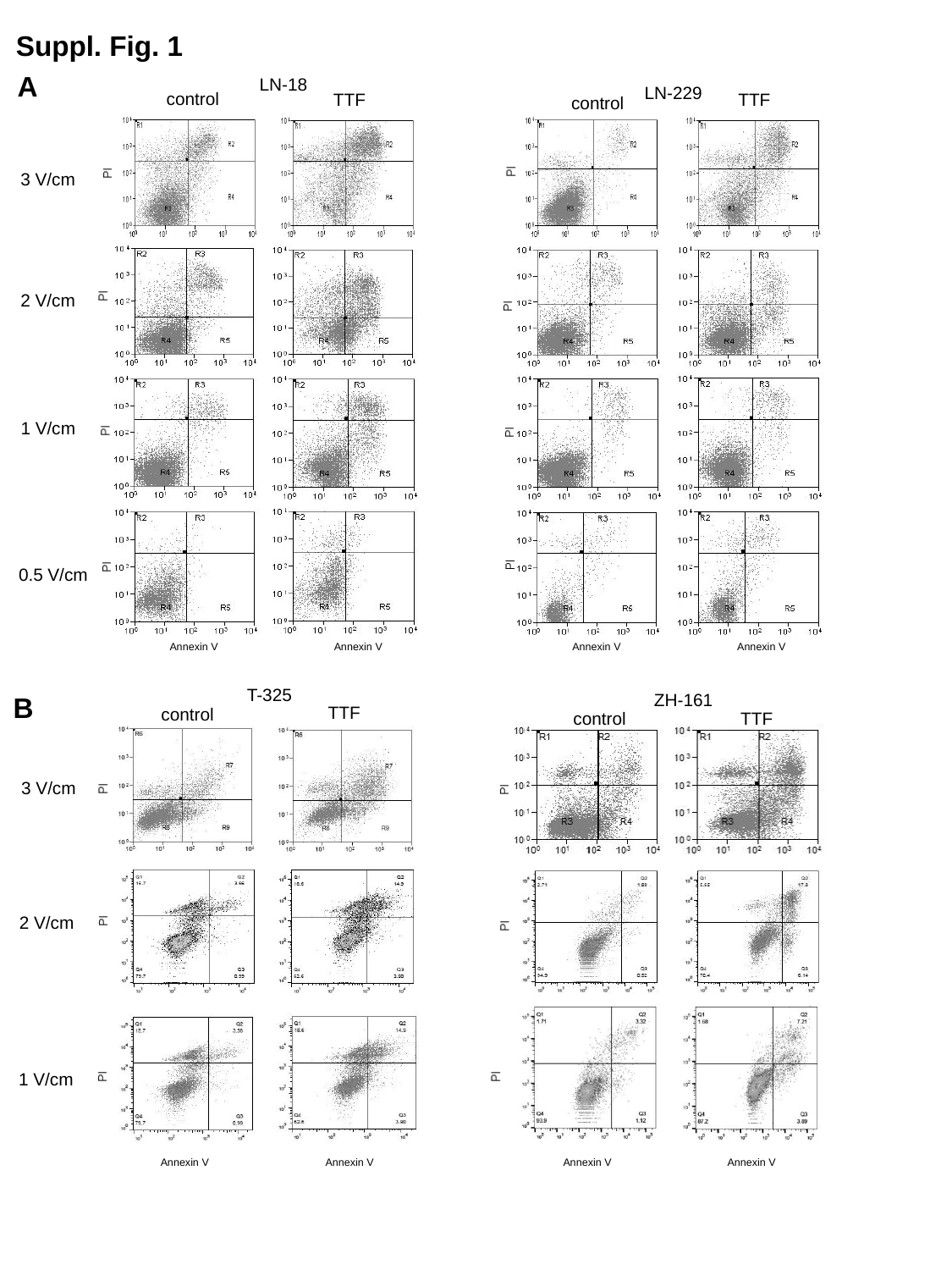

Suppl. Fig. 1 A
LN-18
LN-229
control
TTF
TTF
control
3 V/cm
2 V/cm
1 V/cm
0.5 V/cm
Annexin V
Annexin V
Annexin V
Annexin V
T-325
ZH-161
B
TTF
control
TTF
control
3 V/cm
2 V/cm
1 V/cm
Annexin V
Annexin V
Annexin V
Annexin V

## Slide 2
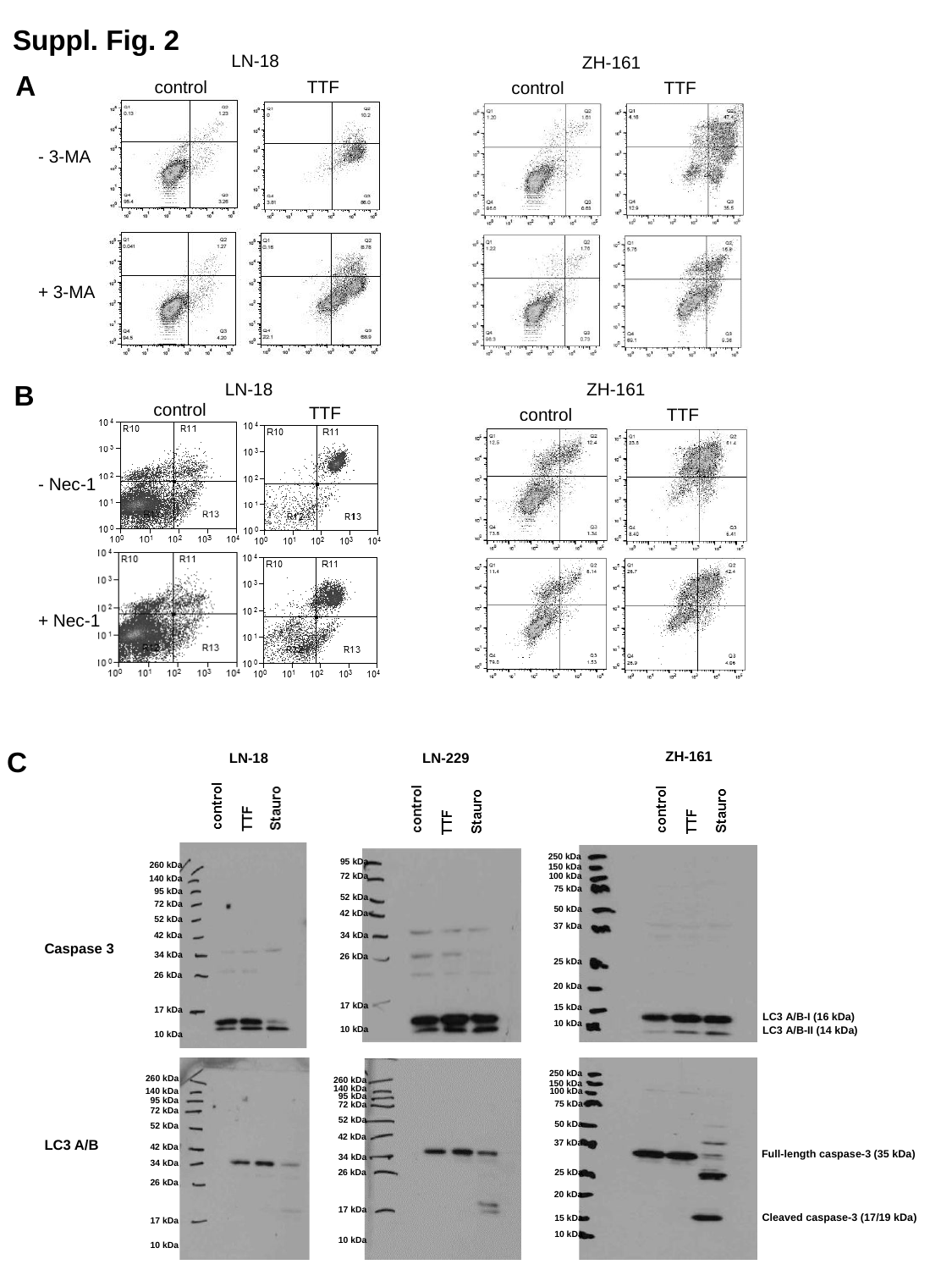

Suppl. Fig. 2
LN-18
ZH-161
A
control
TTF
control
TTF
- 3-MA
+ 3-MA
B
LN-18
ZH-161
control
TTF
TTF
control
- Nec-1
+ Nec-1
C
ZH-161
LN-18
LN-229
250 kDa
95 kDa
260 kDa
150 kDa
72 kDa
100 kDa
140 kDa
75 kDa
95 kDa
52 kDa
72 kDa
50 kDa
42 kDa
52 kDa
37 kDa
42 kDa
34 kDa
Caspase 3
34 kDa
26 kDa
25 kDa
26 kDa
20 kDa
17 kDa
15 kDa
17 kDa
LC3 A/B-I (16 kDa)
10 kDa
10 kDa
10 kDa
LC3 A/B-II (14 kDa)
250 kDa
260 kDa
260 kDa
150 kDa
140 kDa
140 kDa
100 kDa
95 kDa
95 kDa
75 kDa
72 kDa
72 kDa
52 kDa
50 kDa
52 kDa
42 kDa
37 kDa
42 kDa
LC3 A/B
34 kDa
Full-length caspase-3 (35 kDa)
34 kDa
26 kDa
25 kDa
26 kDa
20 kDa
17 kDa
15 kDa
17 kDa
Cleaved caspase-3 (17/19 kDa)
10 kDa
10 kDa
10 kDa

## Slide 3
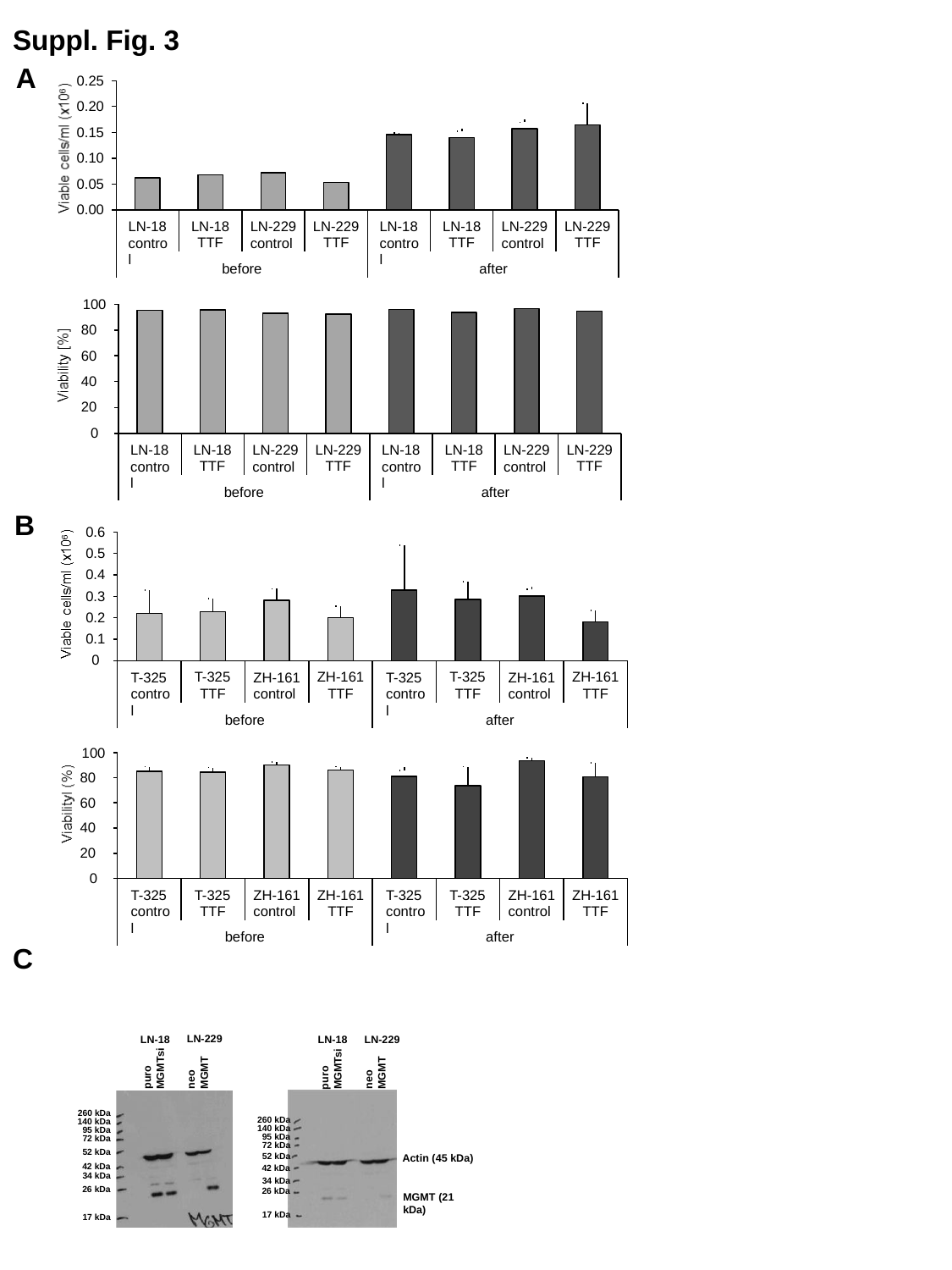

Suppl. Fig. 3
A
0.25
0.20
0.15
0.10
0.05
0.00
LN-18
control
LN-18 TTF
LN-229
control
LN-229 TTF
LN-18
control
LN-18 TTF
LN-229
control
LN-229 TTF
before
after
100
80
60
40
20
0
LN-18
control
LN-18 TTF
LN-229
control
LN-229 TTF
LN-18
control
LN-18 TTF
LN-229
control
LN-229 TTF
before
after
B
0.6
0.5
0.4
0.3
0.2
0.1
0
T-325
control
T-325 TTF
ZH-161
control
ZH-161 TTF
T-325
control
T-325 TTF
ZH-161
control
ZH-161 TTF
before
after
100
80
60
40
20
0
T-325
control
T-325 TTF
ZH-161
control
ZH-161 TTF
T-325
control
T-325 TTF
ZH-161
control
ZH-161 TTF
before
after
C
LN-229
LN-229
LN-18
LN-18
MGMT
MGMTsi
neo
MGMT
puro
MGMTsi
neo
puro
260 kDa
260 kDa
140 kDa
140 kDa
95 kDa
95 kDa
72 kDa
72 kDa
52 kDa
52 kDa
Actin (45 kDa)
42 kDa
42 kDa
34 kDa
34 kDa
26 kDa
26 kDa
MGMT (21 kDa)
17 kDa
17 kDa
