# Supplementary material for: Biological activity of tumor-treating fields in preclinical glioma models
Source: Cell Death Dis. 2017 Apr 20;8(4):e2753–. doi: 10.1038/cddis.2017.171 (PMC5477589; doi:10.1038/cddis.2017.171)
Supplement: Supplementary Figure Legends [file cddis2017171x2.docx]

# Supplementary Figure legends

**Suppl. Fig. 1. TTFields induce cell death in human glioma cells.** A, B. LN-18 (A, left) or LN-229 (A, right), or T-325 (B, left) or ZH-161 (B, right) cells, untreated or exposed to increasing intensities of TTFields as indicated for 48 h, were analyzed for cell death by annexin V/PI staining and data are presented as flow cytometry profiles.

**Suppl. Fig. 2. TTFields-induced cell death involves autophagy and necroptosis.** A, B. LN-18 or LN-229 cells were treated with 3-methyladenine (3-MA, 1 mM) (A) or necrostatin-1 (Nec-1, 100 µM) (B) for 60 min followed by TTFields (2 V/cm or 3 V/cm, respectively) for 72 h. Cell death was assessed by annexin V/PI staining and flow cytometry profiles are shown. C. Whole cell lysates of LN-18, LN-229 or ZH-161 cells, untreated or exposed to TTF (3 V/cm) or staurosporine (1 µM) for 48 h, were analyzed for full-length and cleaved caspase 3, LC3A/B and actin protein levels by immunoblot.

**Suppl. Fig. 3. Effect of TTFields on cell viability during migration and invasion assays.** A, B. LN-18 or LN-229 cells (A) or T-325 or ZH-161 cells (B) were left untreated or exposed to TTFields (2 V/cm) for 24 h. Subsequently, the cells were harvested and adjusted to equal concentrations of viable cells for all conditions. Similar cell counts and viability between cells of the different conditions were confirmed using trypan blue exclusion assay (before). Cells were then seeded for migration or invasion, and in parallel, the remaining cell suspensions were incubated at 37°C and cell counts and viability were assessed by trypan blue exclusion after stopping migration or invasion (after). C. Whole cell lysates of LN-18 puro or MGMTsi, or LN-229 neo or MGMT cells were analyzed for MGMT or actin expression by immunoblot.
